# Karrikins Identified in Biochars Indicate Post-Fire Chemical Cues Can Influence Community Diversity and Plant Development

**DOI:** 10.1371/journal.pone.0161234

**Published:** 2016-08-18

**Authors:** Jitka Kochanek, Rowena L. Long, Allan T. Lisle, Gavin R. Flematti

**Affiliations:** 1 School of Agriculture and Food Sciences, University of Queensland, Brisbane, Queensland, Australia; 2 School of Chemistry and Biochemistry, University of Western Australia, Crawley, Western Australia, Australia; 3 School of Agriculture and Food Sciences, University of Queensland, Gatton, Queensland, Australia; University of California Davis, UNITED STATES

## Abstract

**Background:**

Karrikins are smoke-derived compounds that provide strong chemical cues to stimulate seed germination and seedling growth. The recent discovery in Arabidopsis that the karrikin perception system may be present throughout angiosperms implies a fundamental plant function. Here, we identify the most potent karrikin, karrikinolide (KAR_1_), in biochars and determine its role in species unique plant responses.

**Methods:**

Biochars were prepared by three distinct commercial-scale pyrolysis technologies using systematically selected source material and their chemical properties, including karrikinolide, were quantified. Dose-response assays determined the effects of biochar on seed germination for two model species that require karrikinolide to break dormancy (*Solanum orbiculatum*, *Brassica tourneforttii*) and on seedling growth using two species that display plasticity to karrikins, biochar and phytotoxins (*Lactuca sativa*, *Lycopersicon esculentum*). Multivariate analysis examined relationships between biochar properties and the plant phenotype.

**Findings and Conclusions:**

Results showed that karrikin abundant biochars stimulated dormant seed germination and seedling growth via mechanisms analogous to post-fire chemical cues. The individual species response was associated with its sensitivity to karrikinolide and inhibitory compounds within the biochars. These findings are critical for understanding why biochar influences community composition and plant physiology uniquely for different species and reaffirms that future pyrolysis technologies promise by-products that concomitantly sequester carbon and enhance plant growth for ecological and broader plant related applications.

## Introduction

**Fig 1:**
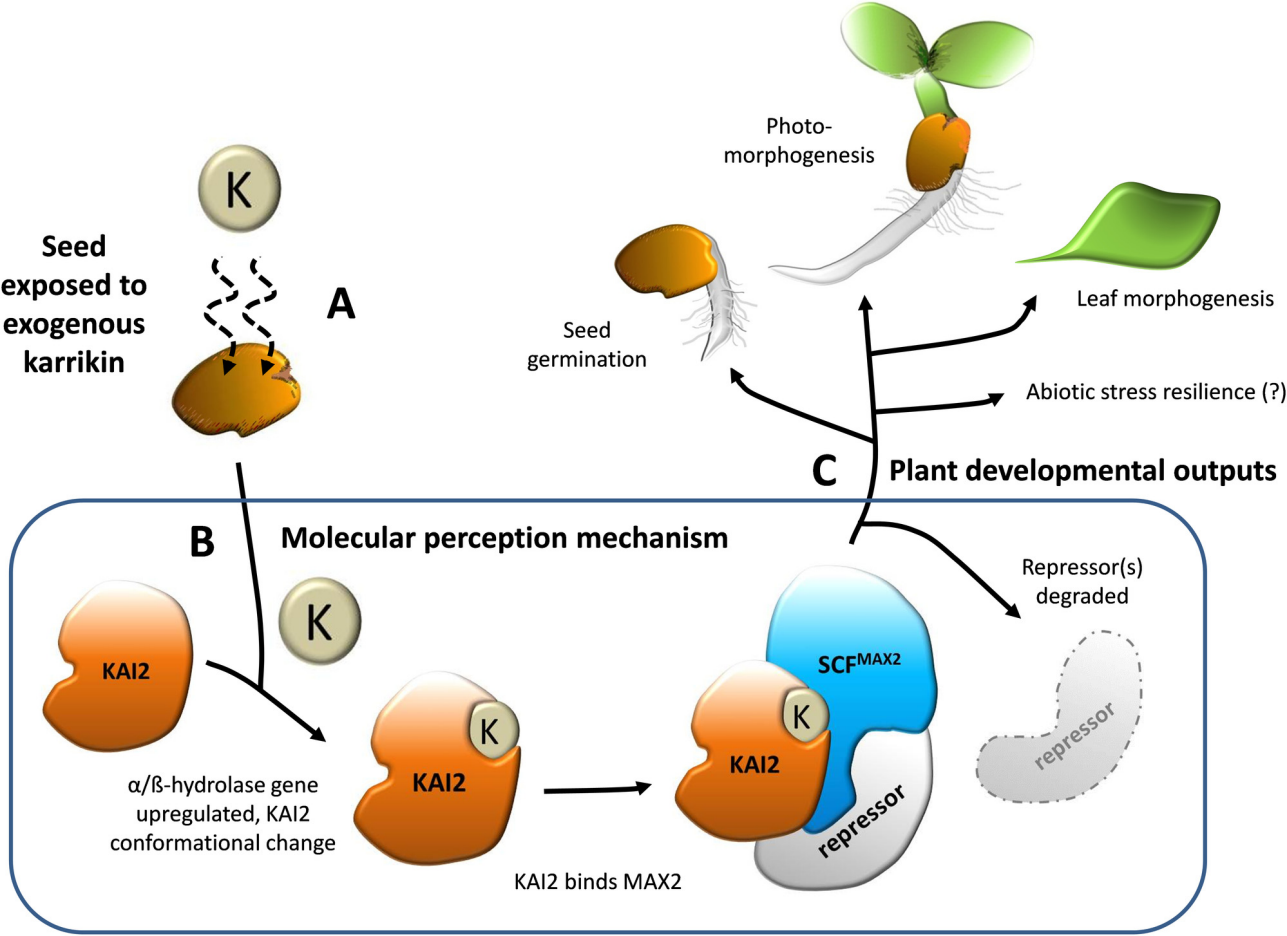
An adaptation of the proposed karrikin signal transduction mechanism in plants. (A) Exposure of seeds to exogenous karrikins is proposed to trigger a molecular response (B) whereby the karrikin binds an α/ß-hydrolase called KAI2 that induces a conformational change in protein shape, thus allowing KAI2 to interact with MAX2, an F-box subunit of an SCF class of E3 ubiquitin-protein ligase complex. SCF^MAX2^ then degrades growth repressing proteins, increasing a seed’s sensitivity to light and stimulating plant developmental processes (C) such as enhanced seed germination, seedling photomorphogenesis, leaf morphogenesis and possibly stress resilience [4,5].

Plants exhibit a unique ability to modify their development and growth as a response to their environmental surroundings, coordinated by endogenous plant hormones [1]. Environmental cues can manifest permissive and inhibitory roles or both can act together to fine-tune plant responses [2]. Karrikins are a family of compounds derived from charred plant materials and smoke that provide chemical cues to stimulate germination from seed banks for fire-following species [3]. In the model plant *Arabidopsis thaliana*, karrikins are proposed to bind a putative α/ß-hydrolase receptor protein, KAI2, transducing a signal to the F-box protein MAX2 that degrades various growth repressing proteins (Fig 1), stimulating seed germination and seedling photomorphogenesis [4]. Critically, recent research indicates the karrikin perception system is fundamental in plant functioning throughout angiosperms [5]. The coincident evolution of vascular plants at the peak of global fire events is a plausible explanation for such widespread adaptive responses and it is hypothesised that the karrikin-induced activity of the KAI2 system mimics a natural pathway initiated by a currently unidentified endogenous substrate or ligand [4,6].

In this present study, we identify the most potent karrikin, karrikinolide or KAR_1_ [7], in biochars and pyrolysis by-products prepared systematically by three distinct technologies, thus drawing parallels between post-fire plant recruitment and biochar mechanisms. We explore biochar because this charcoal-like material, made from pyrolysis of organic biomass under limited or no oxygen conditions, is generating interest globally for having a potentially important role in overcoming the global challenges of climate change through sequestering carbon [8,9] and as a useful soil additive with potential applications in reforestation [10], revegetation [11], soil remediation [12] and agriculture [13,14]. Through examining different pyrolysis technologies and plant-biochar responses, this study reports techniques to produce products from pyrolysis that consistently maximise plant community composition and growth, hence paving the way to overcoming key issues hindering biochar use for plant-related applications.

Pyrolysis has long been part of renewable energy systems, generating syngas and bio-oils to replace fossil fuels, but large scale biochar utilisation is a relatively recent concept (late 1980s) [13]. If sequestered, biochar can provide negative emissions because the thermal transformation stabilises a significant proportion of the carbon into an aromatically-enriched and biologically inert form [13,15]. For ecological and broader plant related applications, research efforts aim to formulate ‘state of the art’ soil and plant enhancing biochars [8], but being a relatively new technology the challenges remain complex [16]. Although elevated cation exchange capacity and organic carbon in biochar-amended soils are strong predictors for positive plant responses [17,18], its use is hindered because the plant-biochar relationship ranges from very positive (+200%) to very negative (-85%) and tends to be biphasic and species specific in dosage-response studies for reasons that remain largely unexplained [14,17,19,20]. To date, hypotheses to explain this biphasic relationship have suggested mechanisms such as the stimulation of beneficial microorganisms at low concentrations and impairment at high [19,21], chemical facilitation or hormesis at low concentrations but phytotoxicity at high [19,22] and increased ethylene production or decreased ethylene oxidation [23]. Another unexplained phenomenon particularly critical for ecological applications is that biochar can alter plant community composition, reducing the density of some species and increasing others within the same site, as observed for Australian native species that were direct-seeded for field reforestation [10] and grassland species sown as a mixture for restoration [11]. It is well documented that wildfires play a critical role in plant ecosystem functioning, altering community composition through mechanisms that include the addition of biologically active compounds, the most potent known permissive compound being karrikinolide [7,24]. Similarly, inhibitory compounds such as trimethylbutenolide, can block seed germination and this interplay of inhibitory and permissive cues is believed to fine-tune seed emergence to coincide with conditions where seedlings are most likely to survive, such as during peak rainfall periods [2,25]. Volatile organic compounds also occur in biochar when liquids and gases re-condense during pyrolysis on the biochar surface and while such compounds have been directly linked to germination suppression and seedling harm [26,27] they have never been linked to plant promotion.

Our research has identified, for the first time, the presence of karrikins in biochar [28] and we quantify karrikinolide in a number of systematically prepared biochars and associate this amount with species specific increases in seed germination and plant size resulting from biochar treatment, drawing parallels between post-fire plant recruitment and biochar mechanisms. Using this systematic approach we provide techniques to produce pyrolysis products into the future that concomitantly sequester carbon and maximise species diversity and plant growth.

## Materials and Methods

### Systematic biochar preparation

**Fig 2:**
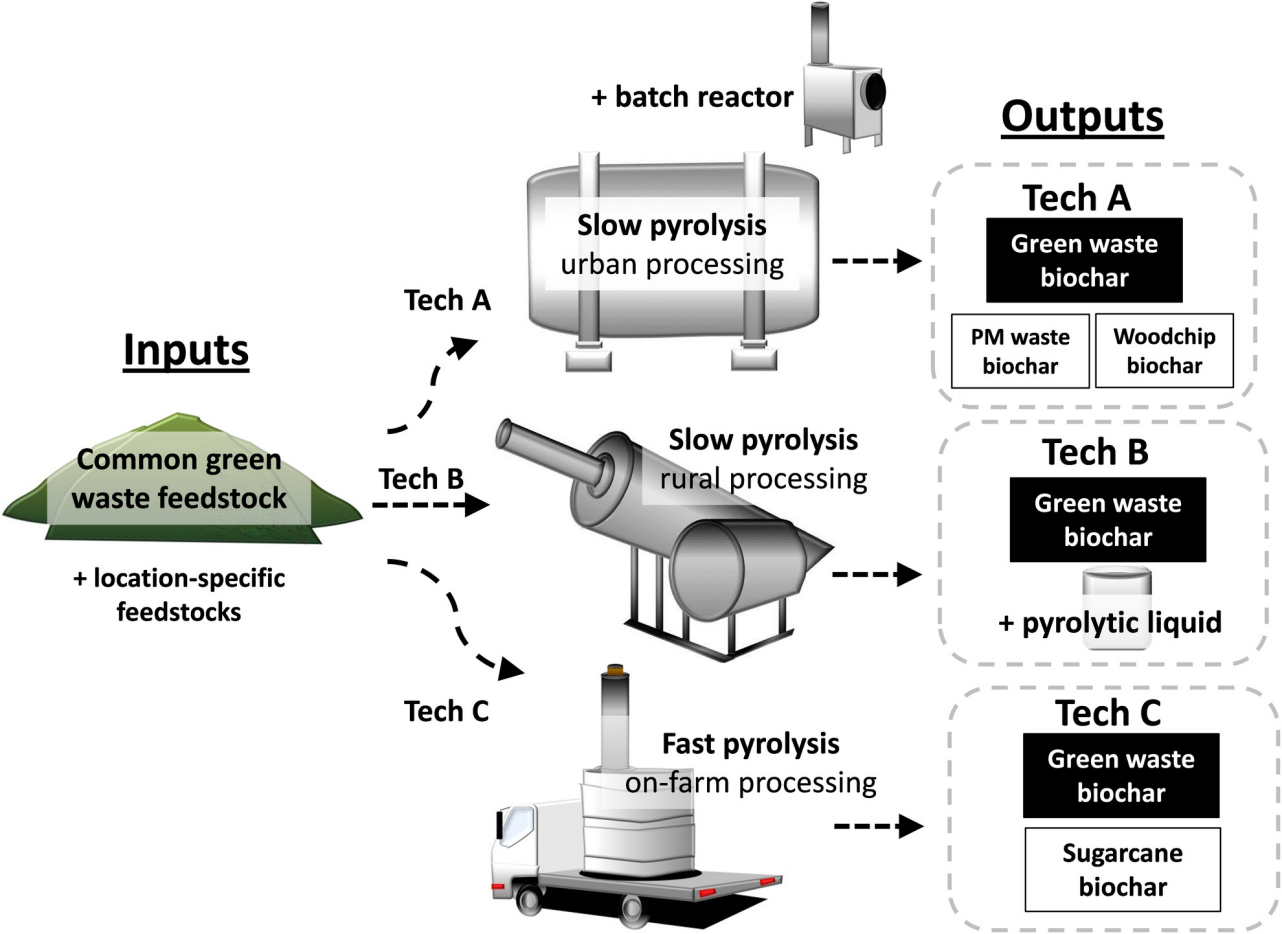
A schematic diagram of the systematic concept used for biochar manufacture. Biochars were made in sizeable quantities by three commercially operating pyrolysis technologies that included a slow pyrolyser designed for high through-put requirements (Technology A, Tech A); a slow pyrolyser for medium volume needs (Tech B) and a mobile fast pyrolyser for on-farm processing (Tech C). Six study biochars were prepared; a common green waste feedstock was made into three biochars by the commercial units, a woodchip and paper mill waste feedstock were made into biochar by a batch reactor version of Tech A (20 L capacity) and sugarcane trash was made into biochar by the commercial Tech C unit to simulate an on-farm feedstock. Additionally, a liquid bi-product was produced by Tech B.

Study biochars were systematically prepared by three commercially operating pyrolysis technologies to align with small to large scale applications (Fig 2, Table 1). Technology A (Tech A) used slow pyrolysis and was a pilot version of a large, centralised plant with a continuous throughput and energy production capacity, applicable to high volume requirements such as urban waste processing. Tech B used slow pyrolysis, with similar conditions to Tech A, but applicable for medium volume requirements such as community or small business undertakings (Table 1; Tech A and B, residence time, 28–40 min; highest heating temperature, HHT, 450–590°C; heating rate, 5–100°C min^-1^). Tech C used fast pyrolysis and was a truck-mountable unit for on-farm processing (residence time, 2–2.5 min; HHT, 600–730°C; heating rate, 500°C min^-1^). Input biomass (feedstock) selection was equally systematic. The raw material manufactured into biochar by all technologies and in sizeable quantities for field-scale experimentation (producing 200 to 1500 kg dw of each biochar) was a woody green waste sized *c*. 20 to 40 mm in diameter and sourced from a common municipal batch, thus allowing direct comparison of biochar characteristics across technologies (the green waste feedstock comprised 49% wood fragments of shredded tree branches, logs and stumps; 43.6% course plant fibres of shredded palm fronds, tree and shrub limbs and prunings; 6% fine plant fibres sized <1 mm; 1.4% removed contaminants such as stones, plastic, paper and cloth). Additional waste biomass inputs gained insights into feedstock effects on biochar properties: sugarcane trash represented a farm waste and was made into biochar (1500 kg dw) by the truck-mountable Tech C unit, while woodchip and paper mill waste were made into biochar using a batch reactor version of Tech A (20 L capacity). Each study biochar was thoroughly mixed prior to storage in closed 240 L plastic bins at 4°C in the dark until use. Further, a liquid by-product was made from Tech B syngas cleaning water (Fig 2). Specifically, the syngas that was not needed to maintain the pyrolysis reaction was cleaned by wet scrubbing through a condenser (using 2–3 misting water jets at 30 L hr^-1^ each) and cooled to *c*. 90°C. Following this process any heavy condensables, such as tars, were precipitated and separated out from the scrubbing water. This water was then re-used to clean charcoal filters through which the scrubbed syngas had been passed for a second cleaning before being collected as the by-product; samples were stored in sealed Schott bottles at 4°C in the dark until use.

**Table 1:** The conditions that prevailed during biochar manufacture. Biochars were made in sizeable quantities by three commercially operating pyrolysis technologies A, B or C or a batch reactor version of Tech A. Inputs were a common green waste feedstock made into biochar by all commercial technologies. Additionally, biochars were made from a sugarcane trash, paper mill waste or woodchip feedstock to expand the study scope.

Biochar feedstock(s)	Green waste			Sugarcane trash	Paper mill waste or woodchip
Technology used	A	B	C	C	A
Type of pyrolysis	slow	slow	fast	fast	slow
Kiln feed rate (kg h^-1^)	300	250	600	600	20 litre batch unit
^1^HHT (°C)	550	590	600–660	680–730	450
Residence time (min)	40	28–29	2.5	2	40
Heating rate (°C min^-1^)	24	75–100	500	500	5–10

^1^HHT is the highest heating temperature.

### Karrikin quantification and chemical characterisation

Karrikinolide was quantitated in the study biochars by first adding 100 ng of ^13^C_5_-labeled KAR_1_ [29] as an internal standard to each of the biochars (100 g). Triplicate samples of each of the biochars were extracted by stirring with ethyl acetate (2 x 150 mL) for 1 hr. The organic solvent was filtered and the combined ethyl acetate extract was evaporated to dryness and dissolved in 50% (v/v) acetonitrile/water (1 mL) and filtered before HPLC separation, followed by GC-MS analysis, as previously described [30]. The mass spectrometer was set to record in selective ion monitoring (SIM) mode using ions *m/z* 121 and 126, which correspond to the base ions of KAR_1_ and ^13^C_5_-labeled KAR_1_, respectively. Comparison of the relative areas indicated the amount of KAR_1_ present in each of the samples. Chemical biochar characterisation was in NATA (National Association of Testing Authorities, Australia) accredited facilities to ISO17025 as previously described [31]. In brief, EC, pH and Colwell phosphorus were analysed by Methods 3A1, 4B1/4B2, 9B2 [32] and total nitrogen was measured by Dumas combustion and exchangeable cations and heavy metals by ICP-AES [33].

### Preliminary plant growth study

**Fig 3:**
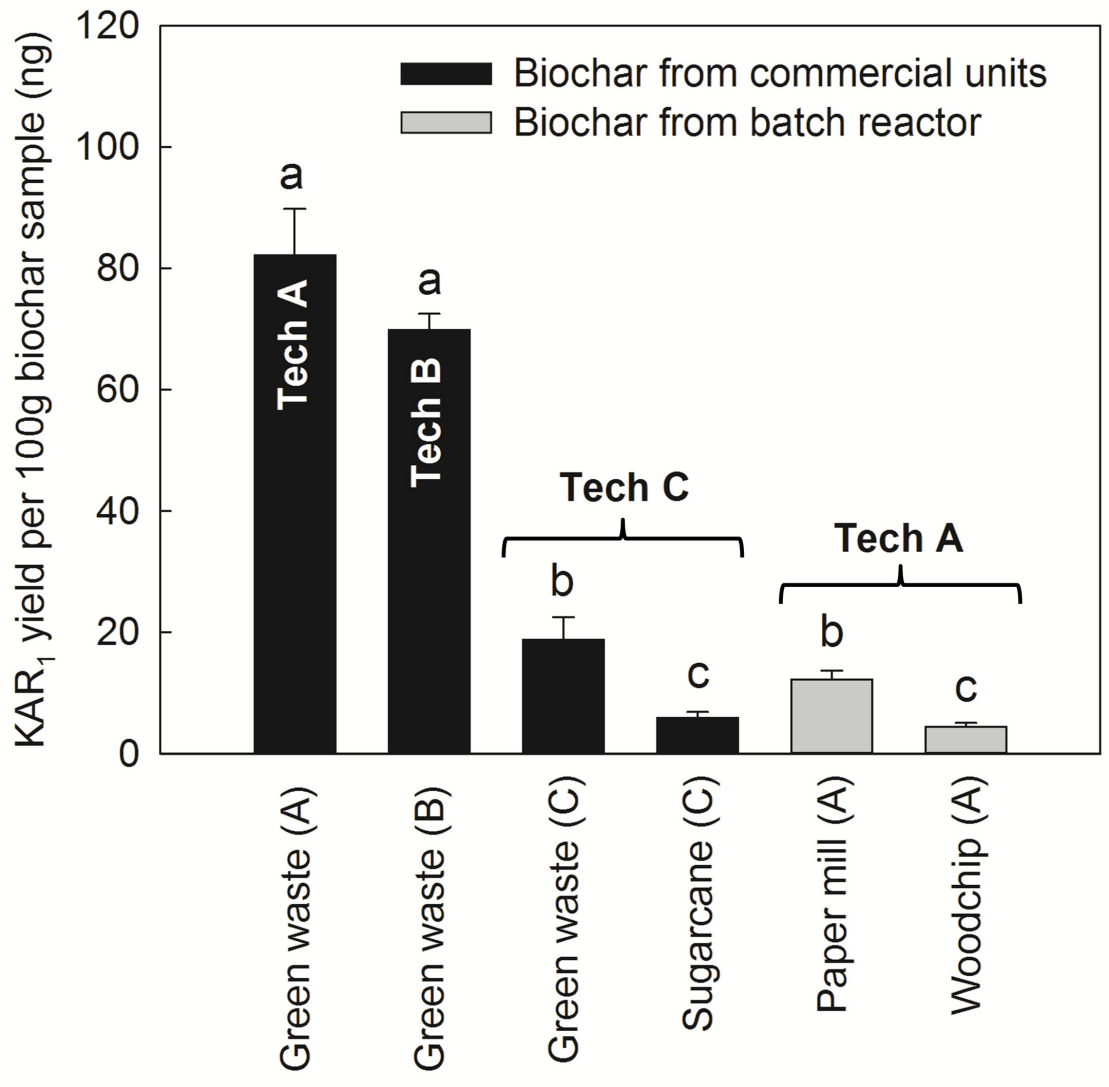
The yield of karrikinolide (KAR_1_) isolated from study biochars as related to pyrolysis conditions and input feedstocks. Biochars were made by Technologies A, B or C in commercial units from a green waste or sugarcane trash feedstock or by a 20 L sized batch reactor using a paper mill waste or woodchip feedstock. KAR_1_ yield is represented as mean ± SEM in each study biochar (ng KAR_1_ per 100 g of biochar, n = 3 replicates). Analysis of variance compared KAR_1_ abundance within different biochars (F_5,11_ = 79.51, *P* < 0.001; data log_10_ transformed prior to analysis); values with different letters are significantly different from one another (Tukey test, α = 0.05).

To begin to understand how study biochars affect plant growth, a karrikin responsive species, tomato (*Solanum lycopersicum* L. cv. Rebel) [34], was grown under glasshouse conditions in media replaced at three rates with a karrikin abundant biochar made from green waste by Tech A (Fig 3; 82.1 ng KAR_1_ yield per 100 g of biochar sample) or a low karrikin biochar made from sugarcane trash by Tech C (5.9 ng KAR_1_/100 g). A factorial design used a biochar type (high or low KAR_1_ content) × biochar rate (0, 3, 10 or 30%) × block combination. One 72 cell tray (each cell of *c*. 50 mL volume sown with one seed) was used per treatment × block combination and trays were maintained in a randomised complete block design using five blocks arranged along a slight light gradient. Control media without biochar used 20 L peat, 6.5 L perlite and 70 g of fertiliser (Nutricote Micro 70 Day, Yates Australia, Padstow, NSW, Australia; nitrogen (N): phosphorus (P): potassium (K) ratio 12.0: 4.4: 8.3, plus trace elements) while biochars replaced the peat at 3, 10 or 30% by volume. One seed was sown per cell to a 0.5 cm depth, covered in vermiculite and maintained under standard glasshouse conditions (25 ± 10°C, natural light) with daily overhead watering. The proportion of germinated seeds was recorded once seedlings were first protruding from media, at 10 d after sowing. Plant growth was quantified at 14 d after emergence, once seedling roots had filled cells and two to four mature leaves had emerged, using five randomly harvested seedlings from each treatment × block combination, at 18 d after sowing. Plant phenotype measurements were hypocotyl length (mm, shoot base to meristem tip), length of the largest leaf (meristem to the leaf tip) and root length. Additionally, above- and below-ground biomass were quantified after drying plant tissues for shoots (mg, hypocotyl and leaves) and roots at 65°C for 48 hr. Media properties determined were bulk density and water holding capacity [35] and EC and pH analysed by Methods 3A1 and 4A1 [32]. Leaf N analysis was performed using an Elementar CHNS analyser and other elements used Inductively Coupled Plasma (ICP) analysis of nitric/perchloric acid digested dried tissue samples.

### Dormancy and plant growth assays

To explore mechanisms behind the observed tomato growth response to biochar, seed dormancy and plant growth of four model KAR_1_ responsive species were investigated in dose-response assays using liquid extracts from all six study biochars. The dormancy assay used two species whose dormancy is readily broken by KAR_1_, *Solanum orbiculatum* [36] and *Brassica tournefortii* [37]. The plant growth assay used lettuce (*Lactuca sativa* L. cv. Archangel Nr) and tomato because both species display plasticity to KAR_1_ [5,34], biochar [19,38] and phytotoxins [39]. The same general treatment structure was used for each trial with biochar type (five and six biochars were compared for the dormancy and plant growth trial, respectively) combined with rate (equivalent to 1, 2.5, 10 and 50% application rates) and a control (Milli-Q water). To compare the effects of pure KAR_1_ on plant parameters, an additional treatment used 0.67 μM KAR_1_ solution (100 μg L^-1^ KAR_1_, the concentration used for screening *S*. *orbiculatum* germination responses to KAR_1_ [40]). Further, the liquid by-product from Tech B (Fig 2) was tested in the dormancy assay, diluted to 1/10 (one part liquid to nine parts Milli-Q water), 1/100, 1/500, 1/1000 and 1/5000 or 1/2000. Thus 27 and 26 individual treatments were used in the dormancy and growth trials, respectively. The experiment was maintained in a randomised complete block design using five blocks (three blocks for *S*. *orbiculatum* due to low seed numbers) arranged along a slight air-flow gradient and each treatment × block combination used one Petri dish. To prepare extracts, 50 g of biochar by dry weight (sieved through a 5.7 mm mesh) was combined with 100 mL of Milli-Q water and rotated in the dark for 24 hours on a vertically mounted wheel. Extracts were vacuum filtered through two Whatman #1 filter papers and a series of extract dilutions gave test solutions: neat (no dilution), 1/5 (one part extract to four parts Milli-Q water), 1/20 and 1/50; equivalent to 500, 100, 25 and 10 t ha^-1^, respectively, at a 10 cm incorporation depth. Bioassays used twenty or five (tomato) seeds sown on two 55 mm diameter filter papers (Filtech, Fairy Meadow, NSW, Australia) within 60 mm diameter plastic Petri dishes and wetted with 1.5 mL Milli-Q water (control), 0.67 μM KAR_1_ or extract solutions [36]. To maintain moisture, Petri dishes were sealed in clear plastic zip-lock bags and for darkness in aluminium foil laminate bags and were incubated at 20 ± 1°C until scoring. The criterion for seed germination was radicle emergence to >2 mm followed by cotyledon expansion. Seeds that remained ungerminated after 14 d were cut open to distinguish empty from full seeds and germination proportion was the number of germinated seeds to the total number of full seeds [41]. The plant phenotype was described for lettuce and tomato at 7 d after sowing [42] for five randomly selected seedlings from each treatment × block combination using shoot length (mm, hypocotyl and cotyledons) and radicle length and, for lettuce, the average number of lateral roots.

### Statistical analysis

Statistical analysis used MINITAB, Release 16 (Minitab Inc., State College, PA, USA). A one-way analysis of variance (ANOVA) compared the concentration of KAR_1_ across study biochars and a *post hoc* Tukey test at the 5% level of significance (α = 0.05) identified differences between means. Glasshouse studies used general linear model (GLM) ANOVA to compare the effects of biochar type (high or low in KAR_1_) and biochar application rate (0, 3, 10 or 30%) and their interactions on plant parameters (germination, plant size and weight). Mean separation was performed by least significant difference (LSD) at α = 0.05. A one-way ANOVA compared the physicochemical properties of propagation media (pH, EC, bulk density, water holding capacity) and a *post hoc* Dunnett’s test (α = 0.05) identified means different to the control. For the seed dormancy and plant growth assays, GLM ANOVA compared the effects of treatments within a species on seed germination or plant size and a *post hoc* Dunnett’s test at α = 0.05 identified means different to the control. Then multivariate analysis (cluster analysis and ordination) simultaneously examined plant indices (shoot and root length for lettuce and tomato, lateral root number for lettuce) and biochar characteristics likely to affect plant growth (pH and EC of extracts, KAR_1_, sodium, nitrogen, potassium and the heavy metals arsenic, chromium and lead; nickel for lettuce). Principal component analysis used singular value decomposition and the first and second principal component were plotted. Across all studies, germination proportion was arcsine and KAR_1_ content of biochars was log_10_ transformed prior to analysis. Homogeneity of variance was met without transformation for other parameters, consequently data are untransformed.

## Results

### Karrikins are systematically quantified in biochar

Karrikinolide was detected in all six study biochars but was abundant only in two (Fig 3). We have previously reported that KAR_1_ can concentrate in biochars [28] and here its quantity was linked to the technology type and unit size used to manufacture biochars. Biochars from the commercial-scale slow pyrolysis units (Tech A and B) contained at least three times more KAR_1_ compared with biochars from fast pyrolysis (slow pyrolysis, *c*. 70–80 ng; fast pyrolysis, *c*. 6–20 ng KAR_1_). We surmise that the hotter and faster pyrolysis conditions in the Tech C unit may have removed or evaporated the KAR_1_, while more was retained in the cooler and slower Tech A and B conditions. For simplicity, the two biochars with >70 ng KAR_1_ are subsequently named ‘karrikin abundant’ or ‘high KAR_1_’ biochars. The size of the unit also affected KAR_1_ retention since biochars from the commercial-scale Tech A unit contained at least seven times more KAR_1_ than biochars from the 20 L sized batch reactor version of Tech A (large unit, *c*. 80 ng KAR_1_; small unit, *c*. 5–12 ng KAR_1_). Highest heating temperature discrepancies between the large and small Tech A units are unlikely to have caused this difference since the large unit was hotter than the small unit (Table 1). A liquid by-product produced by Tech B (Fig 2) was surprisingly abundant in KAR_1_, containing 69 ± 8.8 ng KAR_1_ mL^-1^ (460 nM, *n* = 2). Smoke-water on average contains 265 nM KAR_1_ [3]. The input feedstock did not appear to affect KAR_1_ abundance in biochar as greatly as technology type (Fig 3). For example, both biochars from fast pyrolysis (Tech C) had low KAR_1_ content relative to those from slow pyrolysis (commercial-scale Tech A and B units) despite being from vastly different feedstock sources. However, various other physicochemical differences could be related to input feedstock; the three biochars made from green waste tended to display inferior properties, being more saline and containing more heavy metals, compared to biochars made from other feedstocks (Table 2; Tech B and C green waste biochars exceeded the unrestricted use upper limits for chemical contaminants in composts and mulches, soils or biochar for arsenic, chromium and lead [43–45]). There were no clear trends for macronutrient retention across feedstock or technology used.

**Table 2:** Selected physiochemical properties of study biochars. Biochars were made by Technologies A, B or C in commercial units from a green waste or sugarcane trash feedstock or by a 20 L sized batch reactor using a paper mill waste or woodchip feedstock.

Biochar feedstock	Green waste			Sugarcane trash	Paper mill waste	Woodchip
Technology used	A	B	C	C	A	A
Chemical properties						
EC (dS m^-1^)	2.3	2.7	3.4	1.4	0.5	0.2
pH (CaCl_2_)	8.9	8.8	9.4	8.0	9.0	7.3
N (%)	0.61	0.84	0.47	0.53	0.26	0.26
P (Colwell, mg kg^-1^)	490	990	740	1100	110	18
Exchangeable K (cmol_c_kg^-1^)	15.0	19.0	19.0	8.4	-	1.5
Exchangeable Na (cmol_c_kg^-1^)	5.3	6.6	3.8	0.3	-	0.8
Heavy metals of concern (acid extractable ICP-AES)						
As (mg kg^-1^)	21	30*	32*	<5	<5	<5
Cr (mg kg^-1^)	50	190*	150*	41	67	48
Ni (mg kg^-1^)	6.2	17.0	23.0	23.0	4.9	20.0
Pb (mg kg^-1^)	8.5	72.0*	78.0*	6.4	5.0	<2.0

*Heavy metal concentrations that exceeded the unrestricted use upper limits for chemical contaminants in composts and mulches, soils or biochar (As, 12–500 mg kg^-1^; Cr, 64–1200 mg kg^-1^; Pb, 70–1500 mg kg^-1^ [43–45]).

### Preliminary plant growth study

**Fig 4:**
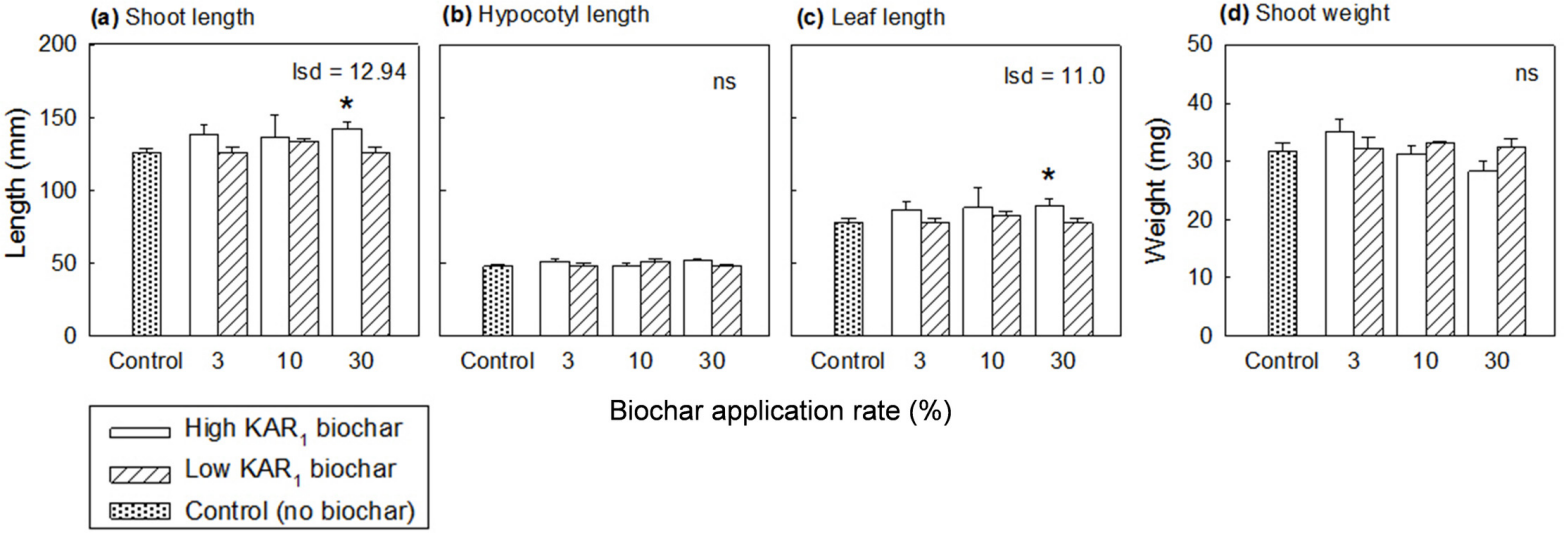
Tomato seedling size in preliminary glasshouse biochar trials at two weeks after germination. Plants were grown in a peat mixture without biochar (control) or with peat replaced at 3, 10 or 30% by either a green waste biochar high in KAR_1_ or a sugarcane biochar low in KAR_1_. All mixtures contained fertiliser to neutralise potential nutritional aspects of biochar [19]. Values represent means ± SEM (*n* = 5 biological replicates). Analysis of variance and means tested the effect of biochar type and rate of application on (a) shoot length; (b) hypocotyl length; (c) length of the largest fully open leaf; (d) average shoot weight per seedling; stars indicate means that are significantly different to the control (lsd, α = 0.05).

The preliminary glasshouse study revealed that media containing high KAR_1_ biochar made from green waste was inferior to the low KAR_1_ biochar made from sugarcane trash, becoming increasingly basic, saline and dense as biochar doses increased (S1 Fig). Conversely, media with the low KAR_1_ biochar remained remarkably similar to peat at all doses. Surprisingly, however, tomato plants became progressively larger the more high KAR_1_ biochar was added to media so that the largest plants grew at the highest biochar dose (Fig 4; with 30% biochar doses plants were 13% larger than from media without biochar), manifested as significantly larger leaves (Fig 4C; 15% longer than the control) with a non-elongated hypocotyl (Fig 4B). Conversely, tomato plants grown in the low KAR_1_ biochar were not substantially larger than the control, hence biochar type determined whether plant architecture was modulated by biochar (S1 Table). Noteworthy is that both biochars resulted in a general decline in leaf nitrogen at increasing doses until plants became deficient (e.g. S2 Fig; tomato leaf tissues contained <2.4% nitrogen, hence were deficient, in media containing 30% high KAR_1_ biochar). By contrast, phosphorus leaf content was largely unaffected by biochar additions (5.5 to 6 g kg^-1^) while potassium concentrations, while healthy (>30 g kg^-1^), showed a trend whereby the high KAR_1_ biochar increased potassium content in leaf tissues (up to 50 g kg^-1^ at 30% biochar) more than other treatments (30 to 35 g kg^-1^).

### Karrikins are linked to germination of dormant seeds

**Fig 5:**
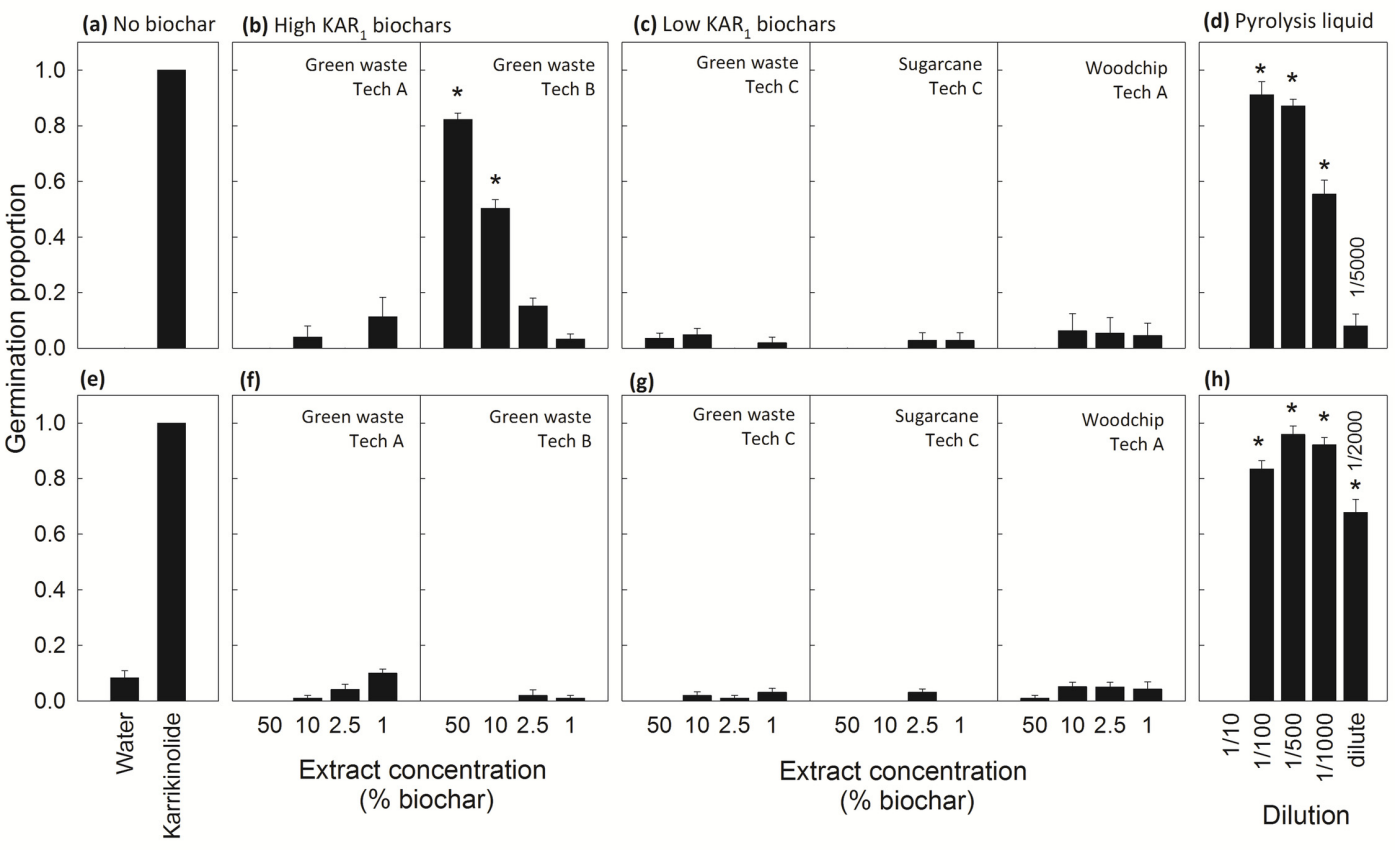
**The seed germination response at 14 d after sowing of two model species that require karrikinolide (KAR**_**1**_**) to break dormancy, *Solanum orbiculatum* (SO, a-d) and *Brassica tournefortii* (BT, e-h), to water, synthetic KAR**_**1**_**, biochar extracts or a liquid by-product from Tech B.** Bars represent mean ± SEM (biological replicates: SO, *n* = 3; BT, *n* = 5). The control (a, e) was Milli-Q water and treatment extracts were prepared from (b, f) karrikin-abundant biochars or (c, g) low-karrikin biochars and (d, h) a liquid by-product of Tech B pyrolysis. Biochar extracts were diluted to concentrations equivalent to 50, 10, 2.5 and 1% biochar application rates and made from green waste by Technologies A, B or C, sugarcane trash by Technology C or woodchips by Technology A. The liquid by-product from Tech B was diluted to 1/10 (one part liquid to nine parts Milli-Q water), 1/100, 1/500, 1/1000 and 1/5000 or 1/2000. Seeds germinated on 0.67 μM KAR_1_ solution (a, e) were not included in analyses (included to confirm seed KAR_1_ sensitivity and viability). Seed germination across all other treatments was compared using analysis of variance within each species (SO, F_25,56_ = 53, *P* < 0.001; BT, F_29,115_ = 143, *P* < 0.001); stars indicate means that are significantly greater than the water control within a species (Dunnett test, α = 0.05). Values were arcsine transformed prior to analysis.

Dose-response assays determined the effects of biochar on seed germination for two model species that require KAR_1_ to break dormancy, *Solanum orbiculatum* and *Brassica tournefortii*. Both species were viable and KAR_1_ sensitive since their seeds germinated to 100% when sown on 0.67 μM KAR_1_ solution compared to 0 and 8%, respectively, when sown on water alone (Fig 5A and 5E). Karrikinolide abundance in biochars was adequate to break seed dormancy but the relationship with seed germination was complex (Fig 5B and 5F). Specifically, one high KAR_1_ biochar successfully germinated *S*. *orbiculatum*, with up to 82% of dormant seeds germinating at the highest biochar dose (Fig 5B, 50% green waste Tech B biochar), compared to 0% of seeds sown on the water control (Fig 5A). As this extract became increasingly diluted, the number of germinated seeds decreased to 50% and below. However, KAR_1_ abundance in biochars did not guarantee seed germination; the dormancy of *B*. *tournefortii* was not broken by either high KAR_1_ biochar (Fig 5F) and *S*. *orbiculatum* seeds were successfully germinated by extracts of the high KAR_1_ Tech B biochar but not the Tech A biochar that contained even higher KAR_1_ concentrations (Fig 5B). Biochars low in KAR_1_ did not improve germination above the control for either species (Fig 5C and 5G). By contrast, the liquid by-product from Tech B stimulated germination almost as effectively as synthetic karrikinolide (Fig 5D and 5H; up to 91% for *S*. *orbiculatum* and 96% for *B*. *tournefortii*), being particularly potent for *B*. *tournefortii*, whereby dilutions from 1/100 to 1/2000 germinated 68% or more seeds.

### Karrikins are linked to shoot elongation

**Fig 6:**
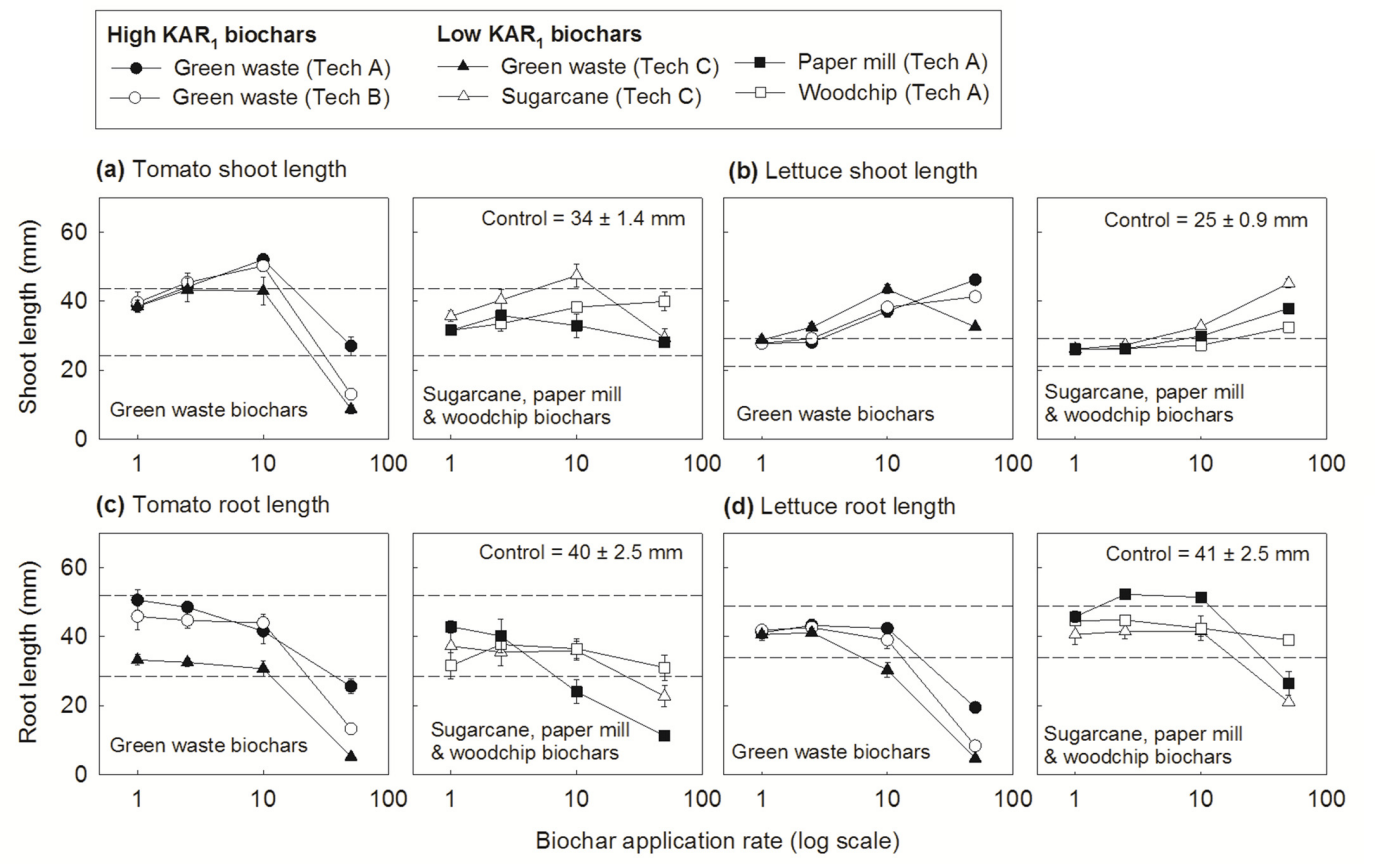
Biochar dosage response curves for tomato and lettuce seedlings at one week after sowing. Biochar extracts were diluted to concentrations equivalent to 1, 2.5, 10 and 50% application rates (shown on a logarithmic scale in a-d) and made from green waste by Techologies A, B or C, sugarcane trash by Technology C, papermill waste by Technology A or woodchips by Technology A. Analysis of variance compared shoot and root length across treatments for (a, c) tomato and (b, d) lettuce (tomato, F_24,95_ = 21.28_shoot_, 18.68_root_, *P* < 0.001; lettuce, F_24,96_ = 46.85_shoot_, 46.23_root_, *P* < 0.001); means above the upper line or below the lower line are significantly different to the control (Dunnett test, α = 0.05).

Dose-response assays determined the effects of biochar extracts on seedling development using tomato and lettuce, species that display plasticity to karrikins. Biochar extracts had a major influence on seedling architecture, but the relationship was unique for each species whereby tomato was generally more sensitive than lettuce to inhibiting elements in biochar at the highest doses. For example, tomato seed germination was generally slowed at the highest biochar doses (S3A Fig; at 3 d after sowing <20% of seeds had germinated compared to *c*. 60% of control seeds when sown on 50% biochar extracts; treatment means were similar to the control at 7 d after sowing, data not shown), while lettuce germination was unaffected by biochar (S3B Fig; there were no significant differences in seed germination at 1 d or 7 d after sowing). Also, tomato plant size generally increased to a maximal peak at intermediate biochar rates (Fig 6A; 2.5 or 10% biochar application rate) and then declined at higher doses so shoots became stunted and small (particularly for 50% green waste biochars) while lettuce plants generally became larger as the biochar dose increased, so that the largest plants grew at the highest dose (Fig 6B; 50% biochar). Nonetheless, the root response to biochar was similar for both species; roots generally became shorter at increasing biochar doses, until they were stunted and burnt at the highest rate (Fig 6C and 6D; 50% biochar).

**Fig 7:**
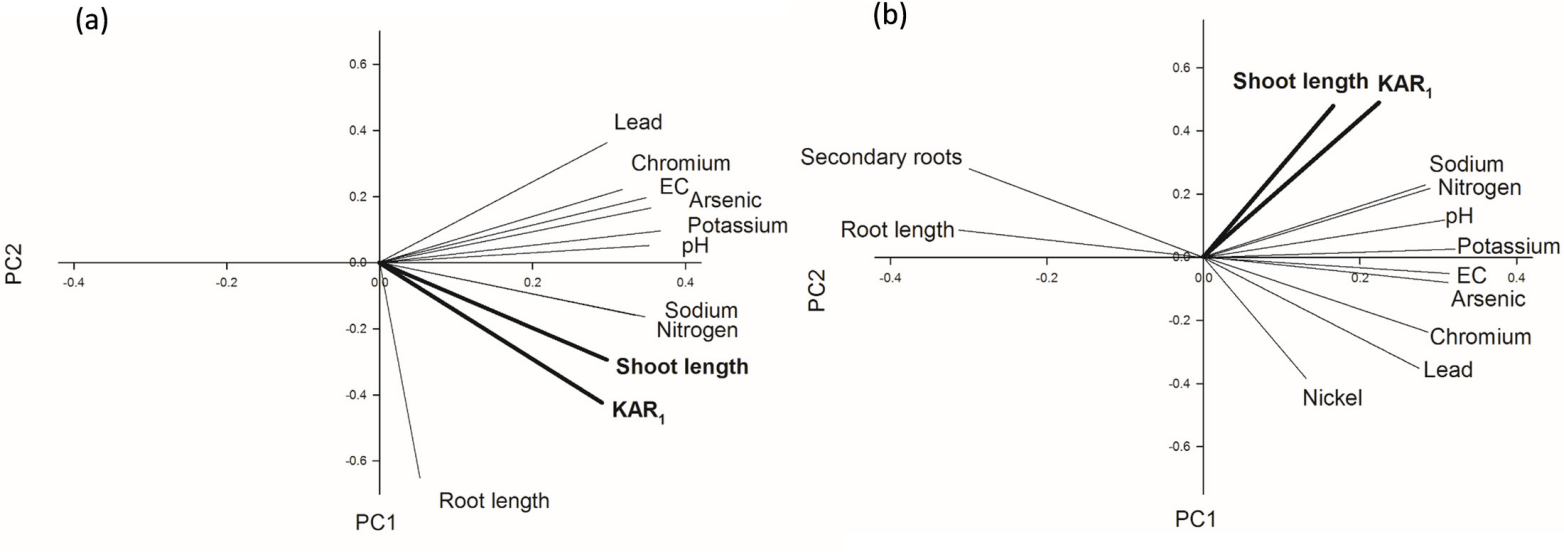
The relationship between the plant phenotype and biochar properities, including karrikin content, for tomato and lettuce seedlings at one week after sowing using multivariate analysis. Multivariate analyses, plotted on principle components 1 (PC1, x-axis) and 2, indicate an association between biochar KAR_1_ content and shoot length for (a) tomato grown on 10% biochar extracts (PC1 accounted for 72.7% and PC2 for 16.9% of the variation) and (b) lettuce on 50% biochar extracts (PC1 = 73.1% and PC2 = 13.8%). At other biochar doses there were no associations between plant phenotype and KAR_1_ content of biochar. Plant phenotype: shoot length, root length (lettuce and tomato), secondary root number (lettuce). Biochar properties: KAR_1_ content, nitrogen, potassium, sodium, EC, pH and heavy metals of concern: chromium, lead, arsenic (tomato and lettuce) and nickel (lettuce). Modelled heavy metals exceeded minimum environmental standards in green waste biochars (Table 2) and/or were at levels that elicit phytotoxicity for the study species [39]. Phosphorus did not change in leaf tissues between treatments (S2 Fig) and substantially reduced the variance explained by the first two components, hence was not modelled.

To begin to understand mechanisms behind such complex plant-biochar interactions, multivariate analysis investigated associations between plant phenotype and biochar characteristics for each species. Importantly, the analysis revealed a relationship between biochar KAR_1_ content and plant size for both species and larger shoots were associated with an increased abundance of KAR_1_ in biochar (Fig 7A and 7B). Critically, however, this relationship was strictly biochar-dose specific: KAR_1_ content and plant size were correlated at the 10% dose for tomato (Fig 7A) and 50% dose for lettuce (Fig 7B), otherwise these vectors were not correlated at other biochar doses. Thus we hypothesise that the phenotypic display to biochar resulted from a species unique sensitivity to chemical stimulants (such as KAR_1_) and inhibitors (such as heavy metals) in biochar. For example, tomato appeared to be responsive to lower KAR_1_ doses than lettuce because tomato shoot size and KAR_1_ were positively associated at lower biochar doses than for lettuce. Conversely, the highest biochar doses were more detrimental to tomato shoot expansion than lettuce, suggesting that tomato was more sensitive to heavy metals or other inhibitors in biochar as doses increased (elevated chromium and lead were associated with stunted tomato shoot and root size at 50% biochar, data not shown; this dose only reduced root growth for lettuce, Fig 7B). A parallel bioassay confirmed this hypothesis; significantly longer shoots were associated with 0.0067–0.067 μM KAR_1_ for tomato and 0.67–6.7 μM KAR_1_ for lettuce (increasing by orders of magnitude, KAR_1_ was tested at 0.0067 to 6.7 μM, unpublished data). Also notable is that for both species karrikin abundance in biochar appeared to minimise negative effects from growth inhibiting elements: green waste biochars were more saline and contained more heavy metals than other biochars but concomitantly grew the largest plants if KAR_1_ was abundant (and the smallest plants where KAR_1_ was low). KAR_1_ abundance in biochars did not influence root size for either species in our study.

## Discussion

Our research is the first to show that the potent plant stimulant, karrikinolide, can concentrate at biologically active levels in biochar. This finding is significant, confirming empirically that KAR_1_ can form by pyrolysis under limited or no oxygen conditions, as well as by the combustion of organic biomass in air, such as during wildfire events [7,30]. Further, our data suggests that karrikin induced plant promotion is a likely mechanism by which biochar can stimulate the germination of dormant seeds and regulate plant development, analogous to post-fire plant recruitment mechanisms that determine community composition. The systematic preparation of study biochars, whereby contrasting technologies and carefully selected feedstocks were used, was fundamental to our understanding of the plant-biochar relationship. Through this unique approach we were able to link KAR_1_ abundance and plant responses to the pyrolysis technology used, showing that biologically active concentrations of KAR_1_ formed under slow, lower temperature pyrolysis and with large feedstock quantities. Conversely, we surmise that most KAR_1_ was consumed or did not form as effectively under fast, higher temperature pyrolysis or from small feedstock volumes and hence was present only at low concentrations in biochars produced under these conditions. It is notable that the chemical reaction that produces karrikins by combustion in air also operates in non-oxidative conditions that produced karrikin abundant biochars in this study. Consequently, oxygen may not be required for karrikin formation in line with previous proposals of its formation from simple carbohydrates [30] and common cellulose combustion products such as pyromeconic acid and propionic acid [3]. Nonetheless, different forms of oxides from compounds in the feedstocks (ultimate analysis showed that the green waste feedstock contained *ca*. 42% oxygen on a dry and ash free basis while the green waste biochars contained 11 to 23% oxygen) and/or entrapped or residual oxygen in the form of air in pores (feedstocks were not N_2_ flushed) may still have contributed to karrikin formation in the pyrolysis technologies used here. Certainly, more research is warranted to understand the precise karrikin-forming reactions involved in both combustion and non-oxidative pyrolysis and karrikin quantities compared from each process when like input feedstocks are used.

Field studies have demonstrated the ability of biochar to alter plant community composition by increasing the seed germination success of some species but inhibiting others (e.g. native Australian species [10], weed species [46]). Using two model species that require karrikins to break dormancy, our study confirmed that certain biochar mechanisms operate via chemical cues. For the Australian native, fire-following species, *Solanum orbiculatum*, we demonstrated that abundant KAR_1_ in one biochar broke seed dormancy. Conversely, germination was suppressed for the weedy *Brassica tournefortii* regardless of karrikin abundance in biochar, possibly via a mechanism analogous to inhibitory compounds in the post-fire environment reversing the karrikin’s effect [2]. Another well documented but unexplained biochar phenomenon is that plant size tends to increase in dose-response trials to a species specific maximal peak and then declines above the peak rate so there is positive plant growth at low to moderate doses and negative at higher doses [17,19,47,48]. One suggested mechanism for such plant responses is chemical inhibitors within biochar inducing facilitation or hormesis at low concentrations but phytotoxicity at high [19]. Here we extend this hypothesis to an interplay of permissive and inhibitory chemical cues as the *modus operandi* by associating the shoot size for two model species that show plasticity for karrikins and phytotoxins, tomato and lettuce, with the abundance of either KAR_1_ or heavy metals within our study biochars, demonstrating that the application rate at which the stimulant or inhibitor dominates the plant size response is species specific. Critically, our results suggest that biologically active concentrations of the karrikin in biochar extended the duration of healthy plant growth under suboptimal conditions (i.e. with increasing heavy metal doses) and indeed karrikins have previously been implicated in enhanced plant resilience to abiotic stress [34, 42, 49], a mechanism that likely evolved to enhance plant establishment in the hostile post-fire environment [5]. Also notable is that karrikin effects on plant development are surprisingly analogous to biochar effects, both being dose-dependent and species specific [5]. To draw further parallels, larger lettuce and Arabidopsis plants observed with increasing biochar doses were linked to an auxin-like upregulation of cell wall genes and upregulation of genes associated with aquaporins [38]. Likewise, karrikinolide has been reported to up-regulate aquaporin-related genes in lettuce [2] and research suggests that auxin-related genes are expressed in the karrikin induced KAI2 pathway for Arabidopsis [5].

Another potentially important discovery from this research was that a by-product of slow pyrolysis, currently a liquid waste, stimulated seed germination and had higher levels of KAR_1_ than traditionally used smoke-water. Specifically, this liquid stimulated the germination of both model dormant species in this study almost as effectively as synthetic karrikinolide (to above 91%), unlike crude smoke-water which often inhibits or delays germination [2]. These differences are perhaps attributable to the karrikin richness of the study liquid, having 460 nM KAR_1_ compared to an average of 265 nM in smoke-water [3], and/or inhibitory compound disparities. Also notable is that this liquid is syngas cleaning water from non-oxidative pyrolysis and is different to ‘pyrolytic liquid’ in the literature (also referred to as ‘pyroligenous acid’ or ‘wood vinegar’) which appears to be made by smoke condensation from oxidative pyrolysis and/or combustion [50,51]. Different again is smoke-water, generated by bubbling smoke from combustion through water [52]. Thus this syngas by-product may be a novel karrikin-rich liquid with potential commercial uses, for example to cost-effectively germinate soil seed banks for ecological rehabilitation or to control weeds in agricultural soils. A unique trivial name, such as syngas-water, may be needed to distinguish it from wood vinegar and smoke-water.

The implications from this study cover broad ecological and plant related themes. Firstly, we demonstrate that biochar mechanisms can operate via chemical cues similar to those in the post-fire environment, thus expanding the understanding of how biochar influences community composition and plant development uniquely for different species. In post-fire ecosystems, karrikins signal the availability of resources, such as nutrients and light, to initiate plant growth [53] and we hypothesise that karrikins in biochar have a similar role, modulating the development of karrikin-responsive plant species as influenced or mediated by other critical factors such as cation exchange capacity, inhibitory compounds and plant-available nutrient concentrations such as potassium [17,18,38]. Secondly, we provide the first steps towards the future development of pyrolysis technologies to create products that consistently stimulate seed germination and plant development. For applications where concomitant carbon sequestration and plant growth are the target, pyrolysis conditions could be optimised to produce karrikin-rich biochars or biochars dosed with synthetic karrikins or karrikin-rich pyrolytic liquids. In fact, biochar may provide a useful medium for field application of ecologically relevant levels of KAR_1_, which is a problem currently hindering progress in this area. Inhibitors to germination, such as free radicals, phenolics and polycyclic aromatic hydrocarbons, have all been implicated in biochar phytotoxicity [26,54] and may impact the effectiveness of karrikin-rich biochars. Hence washing out or quenching biochar inhibitors [26,54] prior to karrikin dosing may be a useful technique, analogous to rainfall washing out inhibitory compounds in the post-fire environment and permitting karrikin-induced seed germination [2]. Finally, the discovery that karrikinolide at biologically active concentrations in biochar extended the duration of healthy plant growth under suboptimal conditions, here with elevated heavy metals, reaffirms that karrikins may have an important role for enhancing plant resilience to abiotic stress. This suggests potentially novel ecological applications of karrikins and biochars for the rehabilitation of spoiled, contaminated or degraded lands.

## Supporting Information

S1 Fig Physicochemical properties of the propagation media used to grow tomato plants in the glasshouse. Plants were grown in a peat mixture without biochar (control) or with peat replaced at 3, 10 or 30% by either a green waste biochar high in KAR_1_ or a sugarcane biochar low in KAR_1_. Media properties, expressed as the mean ± SEM (*n* = 3), are for (a) pH, (b) salinity, (c) bulk density and (d) water holding capacity of the plant growing media. Analysis of variance compared properties across treatments (F_6,14_ = 301_pH_, 617_EC_, 33_BD_, 35_WHC_; *P* < 0.001); means above the critical value line, or below for water holding capacity, are significantly different to the control (Dunnett test, α = 0.05).
(TIF)

S2 Fig Tomato leaf nutrient content. Depicted is the amount of (a) nitrogen, (b) phosphorus or (c) potassium in dried leaf tissues at trial termination (each point is a composite sample, *n* = 1). Points below the ‘deficiency’ line indicate tissue nutrient deficiency and above the ‘healthy growth’ line indicate adequate nutrient content for healthy growth [55].
(TIF)

S3 Fig Tomato and lettuce seed germination at 3 d and 1 d, respectively, after sowing into Petri dishes containing water or extracts of biochar. Biochar extracts were diluted to concentrations equivalent to 1, 2.5, 10 and 50% application rates (shown on a logarithmic scale) and made from green waste by Techologies A, B or C, sugarcane trash by Technology C, papermill waste by Technology A or woodchips by Technology A. Analysis of variance compared seed germination across treatments for (a) tomato and (b) lettuce (tomato, F_24,96_ = 7.53, *P* < 0.001; lettuce, not significant, F_24,96_ = 0.51, *P* = 0.97); means above the upper line or below the lower line are significantly different to the control (Dunnett test, α = 0.05).
(TIF)

S1 Table General linear model analysis of variance and means for tomato plant size and weight. Measurements were at two weeks after germination and tested the effect of a biochar abundant in KAR_1_ or low in KAR_1_ (Biochar type) incorporated into media at four rates (Biochar rate = 0, 3, 10, 30%).
(DOCX)
