# Supplementary figures and images for: Karrikins Identified in Biochars Indicate Post-Fire Chemical Cues Can Influence Community Diversity and Plant Development
Source: PLoS One. 2016 Aug 18;11(8):e0161234. doi: 10.1371/journal.pone.0161234 (PMC4990347; doi:10.1371/journal.pone.0161234)

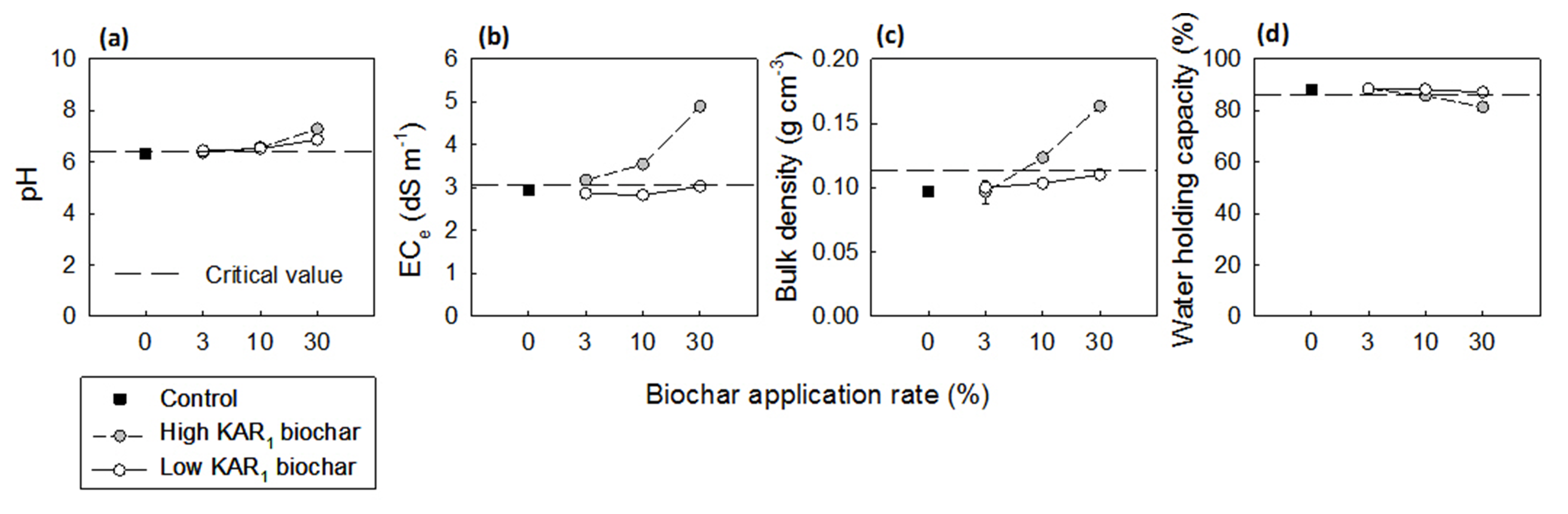

Supplement: S1 Fig — Plants were grown in a peat mixture without biochar (control) or with peat replaced at 3, 10 or 30% by either a green waste biochar high in KAR1 or a sugarcane biochar low in KAR1. Media properties, expressed as the mean ± SEM (n = 3), are for (a) pH, (b) salinity, (c) bulk density and (d) water holding capacity of the plant growing media. Analysis of variance compared properties across treatments (F6,14 = 301pH, 617EC, 33BD, 35WHC; P < 0.001); means above the critical value line, or below for water holding capacity, are significantly different to the control (Dunnett test, α = 0.05). (TIF) [file pone.0161234.s001.tif]

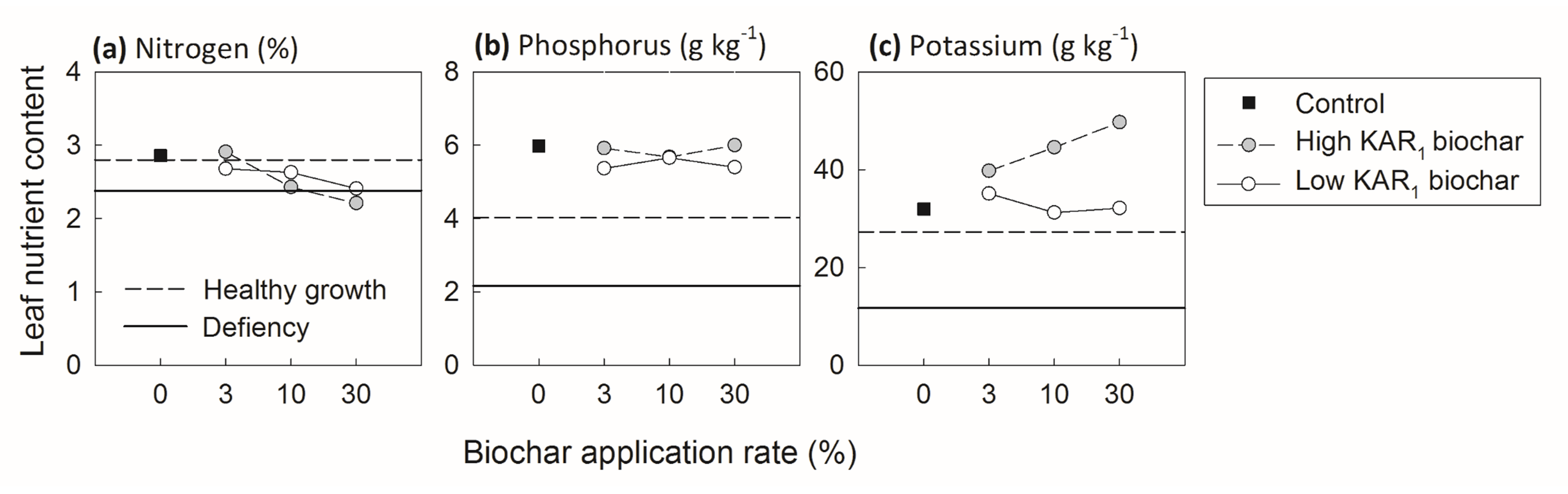

Supplement: S2 Fig — Depicted is the amount of (a) nitrogen, (b) phosphorus or (c) potassium in dried leaf tissues at trial termination (each point is a composite sample, n = 1). Points below the ‘deficiency’ line indicate tissue nutrient deficiency and above the ‘healthy growth’ line indicate adequate nutrient content for healthy growth [55]. (TIF) [file pone.0161234.s002.tif]

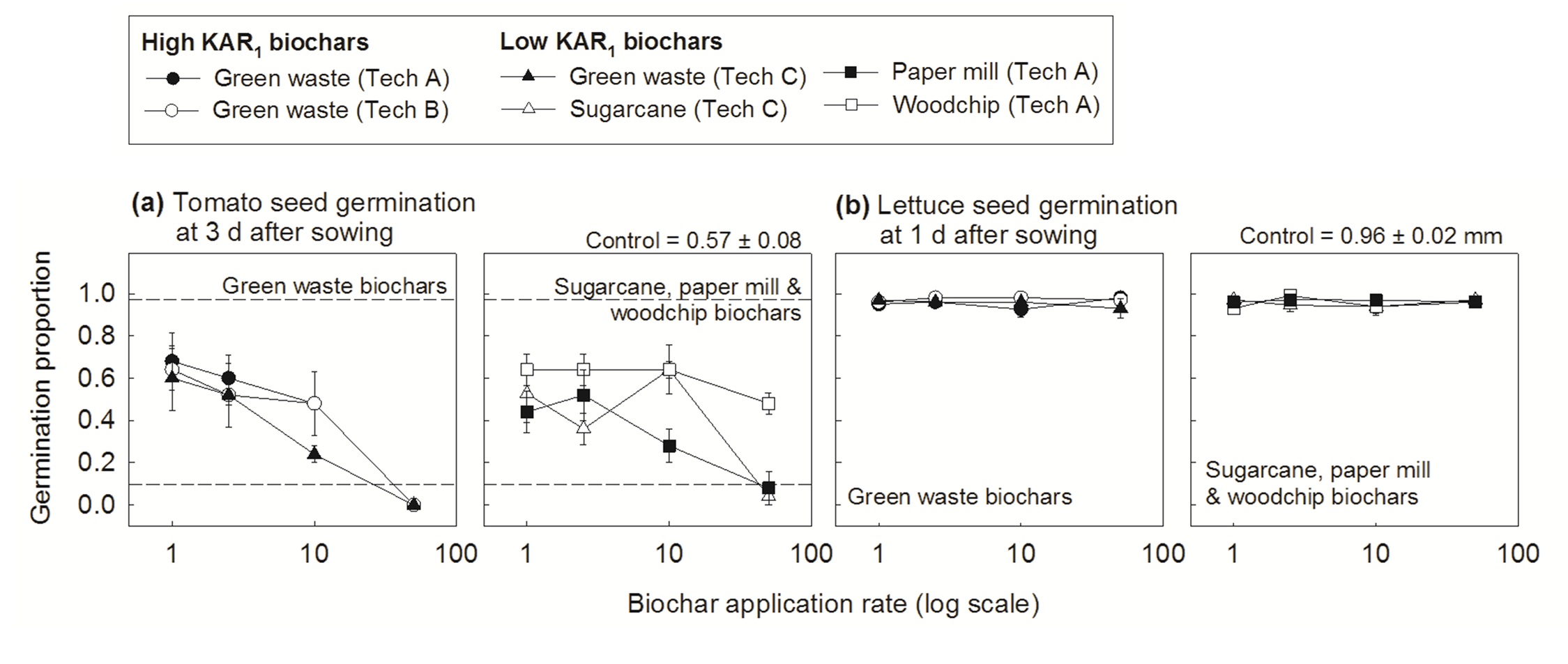

Supplement: S3 Fig — Biochar extracts were diluted to concentrations equivalent to 1, 2.5, 10 and 50% application rates (shown on a logarithmic scale) and made from green waste by Techologies A, B or C, sugarcane trash by Technology C, papermill waste by Technology A or woodchips by Technology A. Analysis of variance compared seed germination across treatments for (a) tomato and (b) lettuce (tomato, F24,96 = 7.53, P < 0.001; lettuce, not significant, F24,96 = 0.51, P = 0.97); means above the upper line or below the lower line are significantly different to the control (Dunnett test, α = 0.05). (TIF) [file pone.0161234.s003.tif]
