# Supplementary material for: Karrikins Identified in Biochars Indicate Post-Fire Chemical Cues Can Influence Community Diversity and Plant Development
Source: PLoS One. 2016 Aug 18;11(8):e0161234. doi: 10.1371/journal.pone.0161234 (PMC4990347; doi:10.1371/journal.pone.0161234)
Supplement: S1 Table — Measurements were at two weeks after germination and tested the effect of a biochar abundant in KAR1 or low in KAR1 (Biochar type) incorporated into media at four rates (Biochar rate = 0, 3, 10, 30%). (DOCX) [file pone.0161234.s004.docx]

|  | Leaf length | Hypocotyl length | Shoot length | Shoot weight | DF |
| --- | --- | --- | --- | --- | --- |
| Block | ns | ns | ns | ns | 4 |
| Biochar type | * | ns | * | ns | 1 |
| Biochar rate | ns | ns | ns | ns | 3 |
| Type × Rate | ns | ns | ns | ns | 3 |

Within each column, nonsignificant differences and significant differences at *P* ≤ 0.05, 0.01 and 0.001 are indicated by ns, *, ** and ***, respectively. Values are the mean of five replicates (one replicate per block, five blocks). Data were not transformed prior to analysis. Residual degrees of freedom was 19.
